# Complete mitochondrial genome of *Liobagrus styani* (Teleostei: Amblycipitidae)

**DOI:** 10.1080/23802359.2016.1275841

**Published:** 2017-01-11

**Authors:** Jia-Yu Huang, Shuai Hu, Xue Bai, E Zhang

**Affiliations:** a Wugang No.3 High School of Wuhan, Wuhan, Hubei Province, P.R. China;; b Wuchang Experimental High School of Wuhan, Wuhan, Hubei Province, P.R. China;; c Institute of Hydrobiology Chinese Academy of Sciences, Wuhan, Hubei Province, P.R. China

**Keywords:** Mitochondrial genome, bullhead torrent catfish, *Liobagrus styani*

## Abstract

The complete mitochondrial genome of *Liobagrus styani* was sequenced by the long and accurate polymerase chain reaction and primer walking sequence method, and each partition was characterized. This genome, with 16,515 bp in length, includes 13 protein-coding genes, 22 tRNA genes, 2rRNA genes, and 2 non-coding regions. Genes encoding on the genome are similar among all vertebrates. These genes except *ND6* and 8 tRNA genes were encoded on the H-strand. Phylogenetic relationship of this species with other congeners was inferred using Bayesian Inference methods based on the genome. Result is contrast to traditional biogeographic explanation for faunal similarity and highlight the need for a further investigation on the formation of allopatric distribution pattern of the genus *Liobagrus* in East Asia.

*Liobagrus styani*, a bullhead torrent catfish of the family Amblycipitidae, was originally described by Regan (1908) but had taxonomically been misidentified in Chinese literature until Wu et al. (2013) who provided a re-description of the fish and clarified its misidentification. So far it occurs only in the Po-He, a tributary flowing to Lake Huanggai in the middle Chang-Jiang basin, at Zhaoliqiao Town, Chibi City, Hubei Province, South China. This fish, with a very narrow area of occupation (less than 10 km^2^), is assessed as critically endangered in the recent species red list of Chinese inland water fish (Cao et al. 2016). The sample of *L. styani* (IHB 2015111503) caught from the Po–He was deposited in the collection of the Institute of Hydrobiology (IHB), Chinese Academy of Sciences. The total genomic DNA was extracted from the pelvic fin preserved in 95% alcohol. Two pairs of long-PCR primer sets were designed for *L. obesus* (Kartavtsev et al. 2007). Fifteen pairs of primer sets were designed for this study.

The complete mitochondrial genome of *L. styani*, with 16,515 bp in length (GenBank accession No. KX096605), includes 13 protein-coding genes, 2 ribosomal RNA (rRNA) genes, 22 transfer RNA (tRNA) genes, and 2 non-coding regions, i.e. the control region (D-Loop) and the origin of L-strand (O_L_). Genes encoding on the genome are similar among all vertebrates (Yu & Kwak 2015). These genes except *ND6* and 8 tRNA genes were encoded on H-strand. Twelve ORF started with ATG and *COX1* with GTG, as found in *L. obesus* (Kartavtsev et al. 2007). For the stop codon, six genes ended with a single base T, *ATP6* with TA, *COX1* and *ND6* with TAG, and *ND1*, *ATP8*, *ND4L* and *ND5* with TAA. Incomplete stop codon was found in the mitochondrial genes of many other fish species (Yu & Kwak 2015). However, the stop codon of *ND6* of *L. obesus* is T––, which is different from that of *L. styani,* and all the other stop codons are the same (Kartavtsev et al. 2007). The base composition of protein-coding genes is T: 27.3%, C: 28.9%, A: 28.4%, and G: 15.4%. The O_L_ was found in the cluster of five tRNA genes (WANCY region) between tRNA^Asn^ and tRNA^Cys^. The D-Loop is 899 bp in length.

Phylogenetic relationships among *L. styani* and other eight congeneric species with complete mitogenome sequences available on GenBank were inferred utilizing Bayesian Inference (BI) methods based on this genome, 12 protein-coding genes (*ND6* was discarded), and 2 rRNA genes. Tree topology (Figure 1) is contrast to traditional biogeographic explanation for faunal similarity shared between Mainland Asia and Korean peninsula plus Japanese archipelago (Li 1981). Under this dispersal explanation, the ancestor of Japanese and Korean species was hypothesized to originate from mainland East Asia during quarternary ice age when these islands were part of mainland; they were isolated from mainland during interglacial period; consequently, the ancestral population speciated into different species occupying Japanese archipelago and Korean peninsula. Evidently, these findings highlight the need for a further investigation on the formation of allopatric distribution pattern of the genus *Liobagrus* in East Asia.

**Figure 1.**
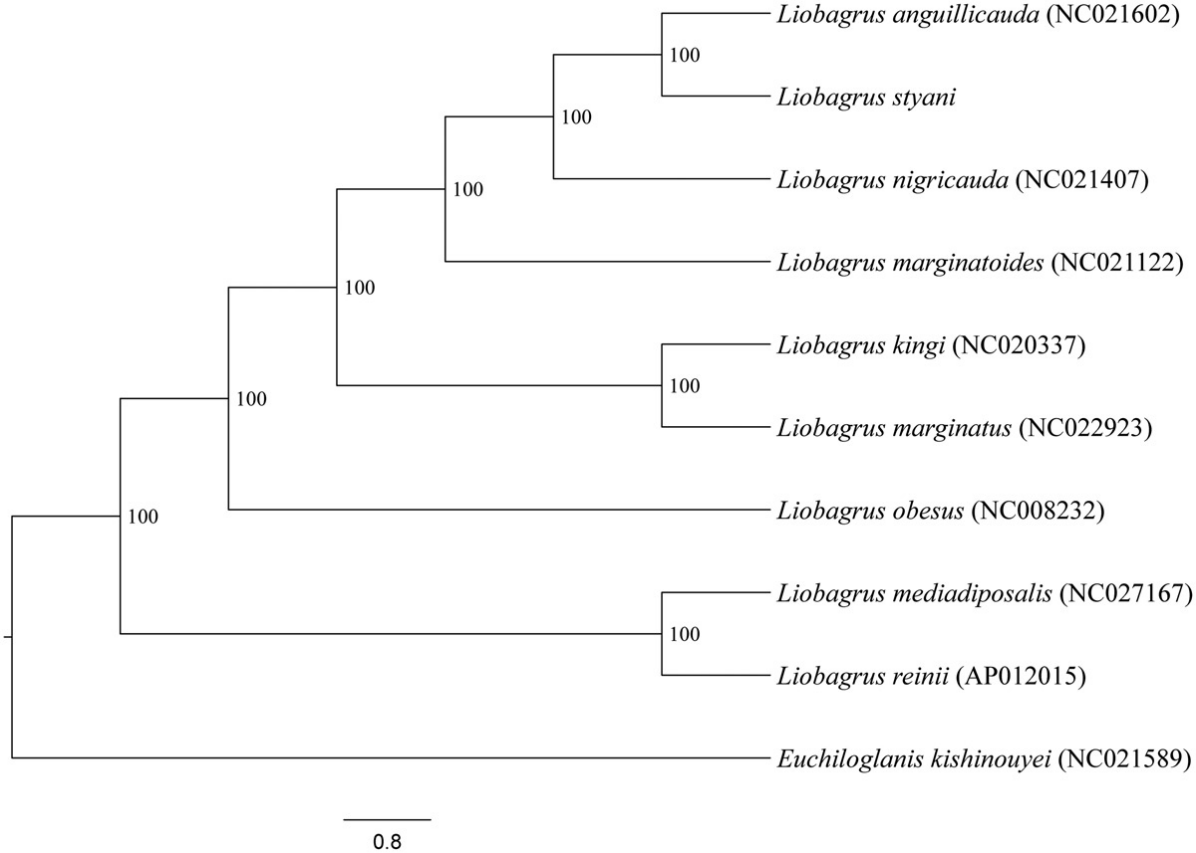
Bayesian Inference phylogenetic tree was constructed using mitogenome sequences. Specimens used for analysis were collected from China, except *L. reinii* (Japan)*, L. mediadiposalis* and *L. obesus* (Korean). *Euchiloglanis kishinouyei* was chosen as outgroup.

## References

[CIT0002] Cao L, Zhang E, Zang C, Cao W. 2016 Evaluating the status of China’s continental fish and analyzing their causes of endangerment through the red list assessment. Biodiversity Sci. 24:598–609.

[CIT0004] Kartavtsev YP, Jung SO, Lee YM, Byeon HK, Lee JS. 2007 Complete mitochondrial genome of the bullhead torrent catfish, *Liobagrus obesus* (Siluriformes, Amblycipididae): genome description and phylogenetic considerations inferred from the Cyt b and 16S rRNA genes. Gene. 396:13–27. 17434693 10.1016/j.gene.2007.01.027

[CIT0001] Li S. 1981 Studies on zoogeographical divisions for freshwater fishes of China. Beijing: Science Press.

[CIT0003] Wu YA, Zhang E, Sun ZW, Ren SJ. 2013 Identity of the catfish *Liobagrus styani* (Teleostei: Amblycipitidae) from Hubei Province, China. Ichthyol Explor Freshw. 24:73–84.

[CIT0005] Yu JN, Kwak M. 2015 The complete mitochondrial genome of *Brachymystax lenok* tsinlingensis (Salmoninae, Salmonidae) and its intraspecific variation. Gene. 573:246–253. 26188159 10.1016/j.gene.2015.07.049

